# Quality Assessment of Platelet-Rich Fibrin-Like Matrix Prepared from Whole Blood Samples after Extended Storage

**DOI:** 10.3390/biomedicines5030057

**Published:** 2017-09-18

**Authors:** Hideo Kawabata, Kazushige Isobe, Taisuke Watanabe, Toshimitsu Okudera, Masayuki Nakamura, Masashi Suzuki, Jietsu Ryu, Yutaka Kitamura, Hajime Okudera, Kazuhiro Okuda, Koh Nakata, Tomoyuki Kawase

**Affiliations:** 1 Tokyo Plastic Dental Society, Kita-ku, Tokyo 1140002, Japan; hidei@eos.ocn.ne.jp (H.K.); kaz-iso@tc4.so-net.ne.jp (K.I.); watatai@mui.biglobe.ne.jp (T.W.); toshiokuderaphd@gmail.com (T.O.); maoh4618@me.com (M.N.); g-yanagidouri-dental@crux.ocn.ne.jp (M.S.); ryu@cap.ocn.ne.jp (J.R.); shinshu-osic@mbn.nifty.com (Y.K.); okudera@carrot.ocn.ne.jp (H.O.); 2 Division of Periodontology, Institute of Medicine and Dentistry, Niigata University, Niigata 9518514, Japan; okuda@dent.niigata-u.ac.jp; 3 Bioscience Medical Research Center, Niigata University Medical and Dental Hospital, Niigata 9518520, Japan; radical@med.niigata-u.ac.jp; 4 Division of Oral Bioengineering, Institute of Medicine and Dentistry, Niigata University, Niigata 9518514, Japan

**Keywords:** platelets, platelet-rich fibrin, platelet-derived growth factor, fibrin fiber, storage

## Abstract

The platelet-rich fibrin–like matrix (PRFM) is usually prepared onsite and immediately used for regenerative therapy. Nonetheless, to meet the clinical necessity of preserving the PRFM without quality deterioration, we developed a method for preparation of PRFMs from short-term-stored whole blood (WB) samples. In this study, to evaluate the practical expiration date of storage, we extended the storage time of WB samples from 2 to 7 days and assessed the quality of the resulting PRFMs. WB samples collected with acid-citrate-dextrose were stored with gentle agitation at ambient temperature. To prepare PRFMs, the stored WB samples were mixed with CaCl_2_ in glass tubes and centrifuged. Fibrin fiber networks, CD41 and CD62P expression, and Platelet Derived Growth Factor-BB (PDGF-BB) levels were examined by scanning electron microscopy (SEM), flow cytometry, and an Enzyme-Linked ImmunoSorbent Assay (ELISA), respectively. Long-term storage had no significant effect on either blood cell counts or platelet functions tested. The resulting PRFMs were visually identical to freshly prepared ones. PDGF-BB levels did not markedly decrease in a time-dependent manner. However, fibrin fibers gradually became thinner after storage. Although the coagulation activity may diminish, we propose that PRFMs can be prepared—without evident loss of quality—from WB samples stored for up to 7 days by our previously developed method.

## 1. Introduction

Among the various types of platelet concentrates, the platelet-rich fibrin-like matrix (PRFM) has been increasingly used as the most convenient biomaterial for regenerative therapy in dentistry [1]. Moreover, this popularity is supported by its multiple functions as both a matrix and scaffold and its higher capacity for tissue regeneration than platelet-rich plasma (PRP) [2,3]. When compared with other platelet concentrate subtypes, PRFM is usually expected to be prepared onsite as per patients’ needs, and immediately used for regenerative therapy. In practice, however, due to a patient’s physical condition or a doctor’s technical capabilities, PRP is extensively prepared on the day or just before a surgical procedure.

In Japan, new regulations for regenerative medicine established in 2014 require all physicians and dentists administering a regenerative therapy that involves a platelet concentrate to record and report the preparation procedures and quality assessment data for PRFM preparations [4]. As a time-saving measure, some physicians or dentists, mainly in private practice, outsource the PRFM preparation process. Therefore, there is a need to develop an off-site PRFM preparation process.

Because anticoagulants, such as citrate and acid-citrate-dextrose (ACD), are added to whole blood (WB) during collection, PRP can be prepared from stored blood and delivered the next day. Even though some physicians or dentists intend to outsource PRFM preparation, due to a lack of anticoagulants, PRFMs cannot be prepared off-site on the next day. Accordingly, another option is to preserve their home-made PRFMs under appropriate conditions. However, there is no reliable scientific evidence to support the safety and effectiveness of a preserved PRFM.

To circumvent this problem, in our previous study [5], we developed a technique for preparation of PRFMs from WB samples stored short-term, and we validated their quality for use as a biomaterial for regenerative therapy. In this previous study, however, we examined WB samples stored only for up to 2 days. It is still unclear how long WB samples can be stored for PRFM preparation without significant quality loss. In blood transfusion, platelet products can be stored for a maximum of 4–7 days, depending on national guidelines and the type of product [6]. Therefore, it can be predicted that platelets may not be useful for medical purposes after this expiry period. In this study, to evaluate biological implications of the officially recommended period of storage for our purposes, we applied our previously developed technique to WB samples stored for relatively long periods (≥5 days) and assessed the quality of the resulting PRFMs.

To help readers correctly understand the identity of the fibrin matrix preparations used in this study, we should emphasize the differences between our PRFM and Choukroun’s PRF: although in a broad sense and judging by visual inspection, our PRFM is almost identical to Choukroun’s original PRF prepared from freshly collected WB samples without anticoagulants, our PRFM may be distinguished from original PRF by the use of both an anticoagulant and CaCl_2_ and the protocol for concentrated growth factors (CGF) preparation in a narrow sense.

## 2. Experimental Section

### 2.1. Blood Collection, Preservation, and Platelet-Rich Fibrin–Like Matrix (PRFM) Preparation

The study design and consent forms for all procedures involving human participants were approved by the ethics committee for human subjects at Niigata University School of Medicine in accordance with the Helsinki Declaration of 1975 (revised in October 2008).

Blood samples (approximately 9.0 mL per tube) were collected from six nonsmoking healthy male volunteers (age 32–68 years) using 21-gauge needles equipped with a conventional vacuum plain glass tube (Plain BD Vacutainer Tube; Becton, Dickinson and Co., Franklin Lakes, NJ, USA) for immediate PRFM preparation or with a vacuum plain plastic tube (Neotube; NIPRO, Osaka, Japan) for stored WB samples as previously described [7,8,9].

For preparing a control PRFM by the conventional method, fresh WB samples were collected into glass tubes in the absence of ACD-A (Terumo, Tokyo, Japan) and were immediately centrifuged by means of a Medifuge centrifugation system (Silfradent S.R.L., Santa Sofia, Italy). This centrifuge was designed to prepare CGF (which may be considered a member of the PRF family) and employs a program that automatically changes the centrifugal speed as follows: 30’’, acceleration; 2’, 2700 rpm (600× *g*); 4’, 2400 rpm (500× *g*); 3’, 3000 rpm (800× *g*); and 36’’, deceleration and stop [10].

For delayed preparation of PRFM, WB samples were collected into plastic tubes in the presence of ACD-A and stored for up to 7 days at ambient temperatures (20–24 °C) with gentle agitation using a tube rotary mixer (NRC-20R; Nissin, Tokyo, Japan). At various time points, the stored WB samples were transferred into glass tubes, warmed at 37 °C, intermittently mixed with 200 μL (20 μL × 10 times) of a 10% CaCl_2_ solution and centrifuged on the Medifuge centrifugation system. After elimination of the red blood cell (RBC) fractions by forceps, the resulting PRFM samples were immediately compressed with a stainless-steel PRFM compression device (PRF stamper^®^; JMR Corp. Ltd., Niigata, Japan) [11] and washed thrice with PBS for scanning electron microscopy (SEM) or stored without washing at −80 °C until determination of PDGF-BB levels.

### 2.2. Measurement of Glucose and Ca^2+^ Levels and pH

Prior to Ca^2+^ addition, the stored WB samples were quickly centrifuged at 415× *g* for 3 min to obtain the plasma fraction, which was used to determine total free Ca^2+^ levels by means of a commercial kit based on the MXB method (Calcium E-test Wako; Wako Pure Chemicals, Osaka, Japan) as described elsewhere [5].

For PRFM preparation, the supernatant serum fractions obtained after centrifugation were subjected to analysis of Ca^2+^ levels as described above and to quantification of glucose with a commercial kit based on the GOD method (Glucose CII Test Wako; Wako Pure Chemicals) [5]. The serum fractions were also subjected to measurement of pH with pH indicators (MColorHast; EMD Millipore Corp., Billerica, MA, USA) [5].

### 2.3. Quantification of a Growth Factor by an Enzyme-Linked Immunosorbent Assay (ELISA)

PDGF-BB levels were measured in the PRFM extracts using the Human PDGF-BB Quantikine ELISA Kit (R&D Systems, Inc., Minneapolis, MN, USA) as previously described [8,11,12]. In brief, individual PRFM samples were minced and homogenized for 1 min with sample tube size disposable homogenizers (BioMasher II; Nippi, Tokyo, Japan). After centrifugation, the resulting supernatants were analyzed by an ELISA.

### 2.4. Determination of Blood Cell Counts

The total number of blood cells in WB samples and in fractionated liquid samples was determined in the same types of sample tubes and an automated hematology analyzer (pocH-100iV Diff; Sysmex, Kobe, Japan) [5,13]. RBCs, white blood cells (WBCs), and platelets were counted either immediately after blood collection or after storage, but before centrifugation.

### 2.5. Flow-Cytometric (FCM) Analyses

The platelet fraction was isolated from WB samples by centrifugation (530× *g*, 10 min), washed twice with PBS, and resuspended in PBS at a density of 1–2 × 10^8^/mL. The platelets were incubated with 10 mM adenosine 5’-diphosphate (ADP; Wako Pure Chemical, Osaka, Japan) or 0.1% CaCl_2_ (Wako) for 15 min at ambient temperature. To stop the reaction, an equal volume of a commercial fixative, ThromboFix (Beckman-Coulter, Brea, CA, USA) was added to each platelet suspension (100 μL) and incubated for 30 min. Platelets were then washed twice with PBS and probed with both a phycoerythrin (PE)-conjugated mouse monoclonal anti-CD41 antibody and a fluorescein isothiocyanate (FITC)-conjugated mouse monoclonal CD62P antibody (1:20) (BioLegend, San Diego, CA, USA) for 45 min at ambient temperature. After two washes with PBS, platelets were analyzed on a flow cytometer (Cell Lab Quanta SC; Beckman-Coulter Inc., Brea, CA, USA) as previously described [14]. For isotype controls, mouse IgG1 (BioLegend) was employed.

### 2.6. Scanning Electron Microscopy

To examine the microstructure of fibrin fiber networks, PRFM samples were compressed, washed thrice with PBS, and cut into small pieces. Then, the PRFM pieces were fixed with 2.5% glutaraldehyde, dehydrated with a series of ethanol and t-butanol washes, freeze-dried, and finally examined by SEM (TM-1000, Hitachi, Tokyo, Japan) with accelerating voltage 15 kV, as previously described [5,15].

### 2.7. Evaluation of Platelet Surface Antigen Expression by an Immunofluorescence Assay

Platelet concentrates were prepared from stored WB samples, rinsed, and resuspended in PBS in sample tubes. Platelets were then treated with CaCl_2_ at a final concentration of 0.1% and incubated for 15 min at ambient temperature. ADP (10 mM) served as a positive control [16]. After completion of the required incubation time, the reaction was stopped by addition of ThromboFix (Beckman Coulter Inc., Brea, CA, USA). The platelets were washed twice and incubated with anti-human CD41 or CD62P monoclonal antibodies (1:20; BioLegend, San Diego, CA, USA) (primary antibodies) for 40 min at ambient temperature. Next, the platelets were again washed twice with PBS and were probed with a secondary antibody, a goat anti-mouse IgG H&L antibody (an Alexa Flour^®^ 555 conjugate; 1:50; Abcam, Cambridge, MA, USA), for 30 min at ambient temperature. Finally, after subsequent PBS washes, the platelets were mounted with an antifade mounting medium (Vectashield^®^; Vector Laboratories, Burlingame, CA, USA), and CD41 and CD62P expression levels were examined under a fluorescence microscope equipped with a cooled CCD camera (Nikon, Tokyo, Japan).

### 2.8. Statistical Analysis

The results are reported as mean ± standard deviation (SD). For multigroup comparisons, statistical analyses were performed by one-way analysis of variance (ANOVA) (SigmaPlot 12.5; Systat Software, Inc., San Jose, CA, USA) with Bonferroni’s post hoc test. Differences with *p*-values < 0.05 were considered statistically significant.

## 3. Results

### 3.1. Time-Dependent Changes in The Characteristics of Whole Blood Samples

**Figure 1:**
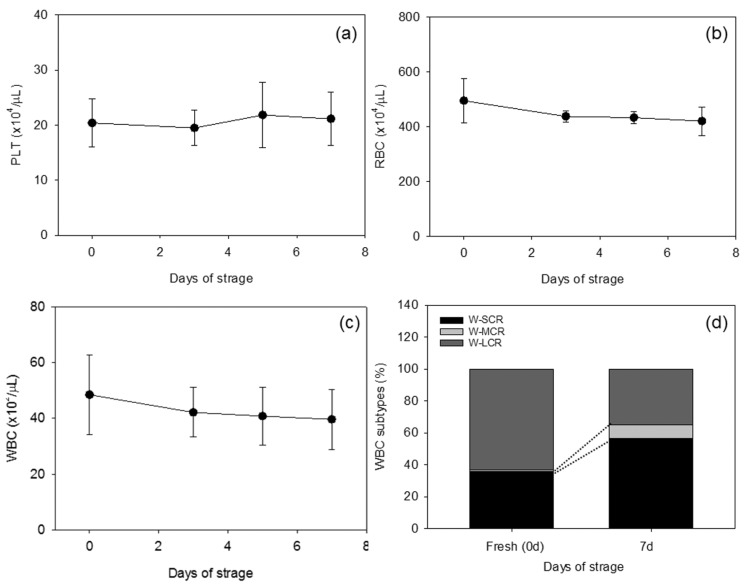
(**a**–**c**) Stable counts of platelets, red blood cells (RBCs), and white blood cells (WBCs) in stored whole blood samples (*n* = 8); (**d**) A comparison of WBC components between fresh and 7-day-stored WB samples. The data were calculated from an average of 8 samples. W-SCR: WBC small cell ratio, W-MCR: WBC middle cell ratio, W-LCR: WBC large cell ratio.

WB samples were stored with gentle agitation at ambient temperature because they were collected into plain plastic tubes and stored for up to 7 days. During this period, both the platelet and RBC counts did not change significantly (Figure 1a,b). Additionally, WBC counts did not shift, but relative percentages of WBC subtypes underwent marked alterations (Figure 1c,d). The percentages of small and medium-size components of WBCs, such as lymphocytes, increased, whereas those of the large components, such as granulocytes, decreased.

**Figure 2:**
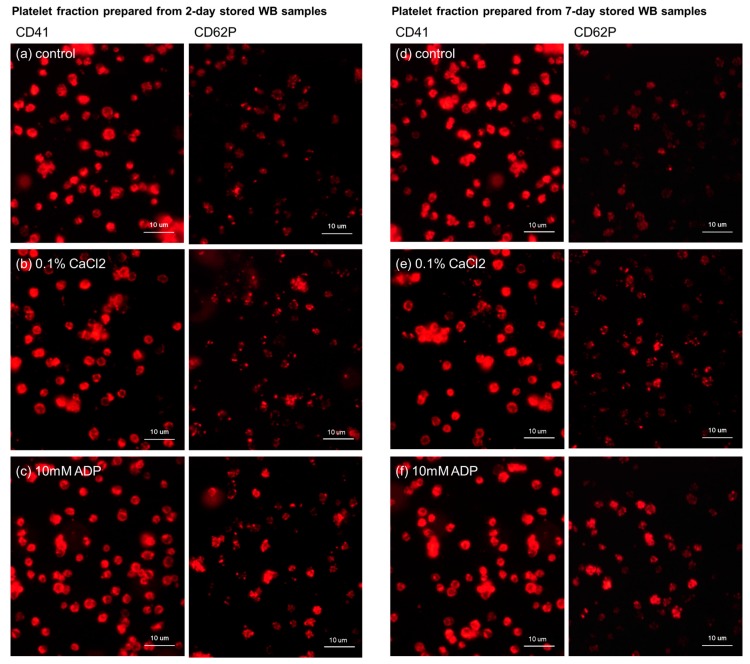
Immunofluorescent staining of CD41 and CD62P expressed in platelets isolated from 2-day- or 7-day-stored WB samples. (**a**,**d**) Control resting platelets; (**b**,**e**) platelets stimulated by 0.1% CaCl_2_ for 15 min; and (**c**,**f**) platelets stimulated by 10 mM ADP for 15 min. The platelets were derived from the same donor and were distributed with almost the same density in all the dishes (views).

Platelets’ responses to stimulants were evaluated by comparing the expression of CD62P with that of CD41 [17]. After storage for 2 days, CD41 expression was similar among all the samples, regardless of the external stimuli (0.1% CaCl_2_ or 10 mM ADP for 15 min; Figure 2). In contrast, CD62P expression levels were upregulated by the CaCl_2_ or ADP challenge. The 7-day storage duration did not alter the platelet activation responses. CD62P expression levels were likewise increased by treatment with similar concentrations of CaCl_2_ and ADP.

**Figure 3:**
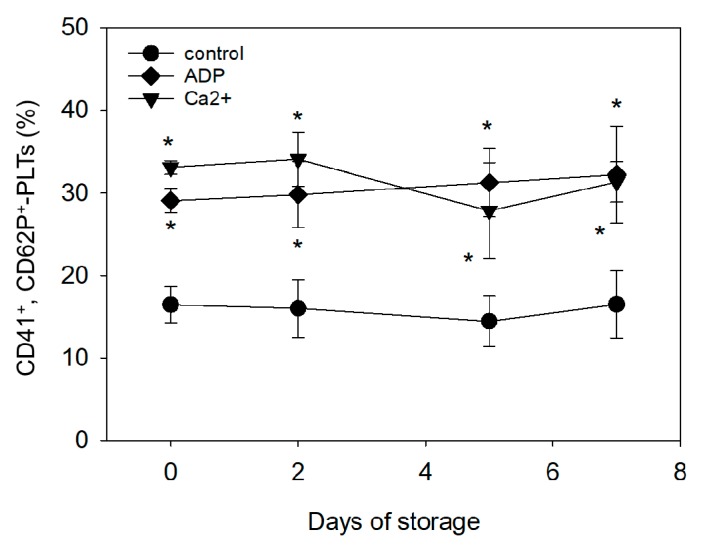
Flow-Cytometric (FCM) analysis of CD41- and CD62P-double-positive platelets in platelet fractions that were prepared from fresh or stored WB samples and stimulated with 10 mM ADP or 0.1% CaCl_2_ for 15 min (*n* = 4). * *p* < 0.05 as compared with control platelets at the same time points. No significant differences were observed in time-course changes.

Similar observations were made during quantitative FCM analysis (Figure 3). In terms of elevated CD62P expression levels, platelets’ responsiveness to ADP or CaCl_2_ stayed at constant levels with storage time.

**Figure 4:**
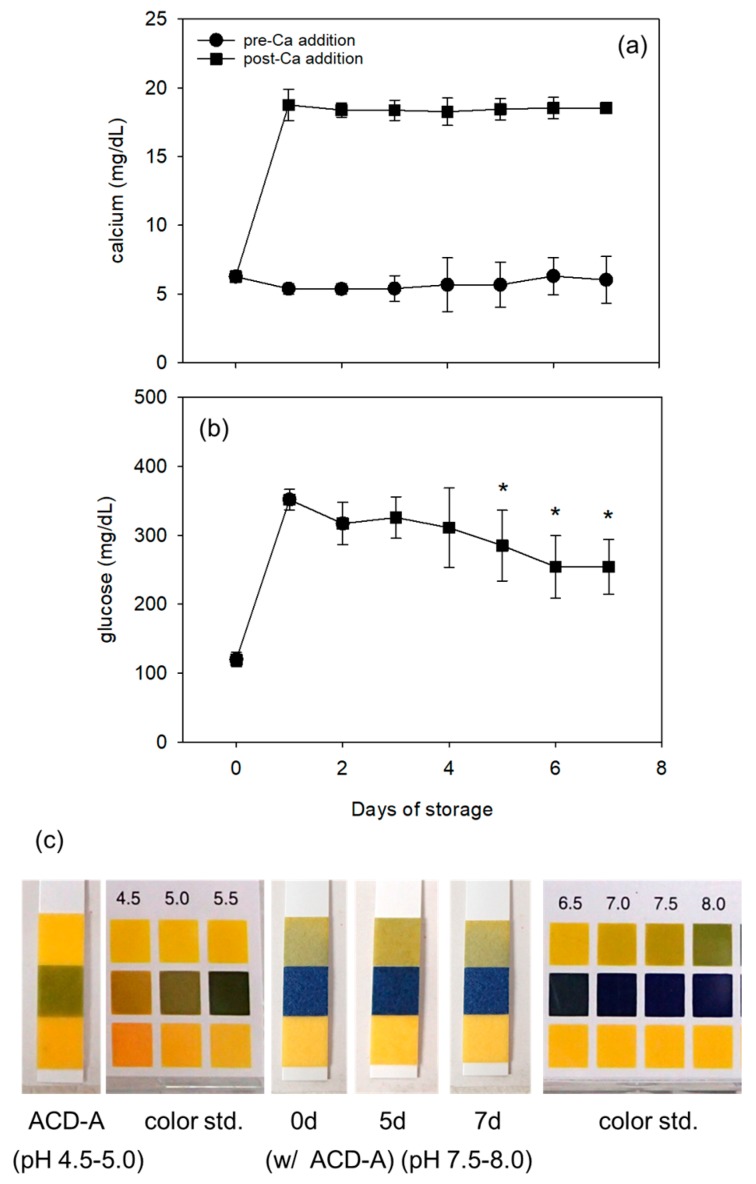
Stable Ca^2+^ (**a**) and glucose levels (**b**) and pH (**c**) of fresh and stored WB samples. Because stored WB samples contained ACD-A as an anticoagulant, CaCl_2_ was added to the samples for PRF clot formation. Ca^2+^ levels were determined before and after the addition of CaCl_2_. Glucose levels were determined in WB samples before the addition of CaCl_2_. * *p* < 0.05 as compared with the individual control levels on day 1 (*n* = 8).

In the liquid fraction of WB samples, Ca^2+^ levels remained similar throughout the storage period, whereas glucose levels, mostly increased by ACD-A, decreased with storage time (Figure 4a,b). Plasma pH stayed at 7.5 ~ 8.0 (Figure 4c).

### 3.2. Time-Dependent Changes in the Quality of The Resultant PRFM Samples

**Figure 5:**
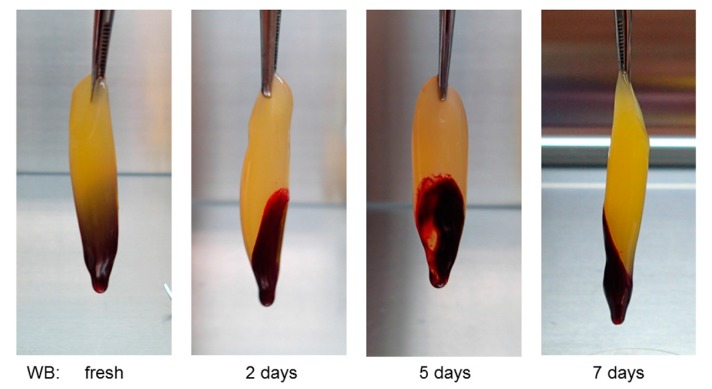
Visual appearance of platelet-rich fibrin–like matrixs (PRFMs) prepared from WB samples stored for the indicated periods. WB samples were simultaneously collected from the same donor. Similar PRFM samples were obtained from three other experiments.

**Figure 6:**
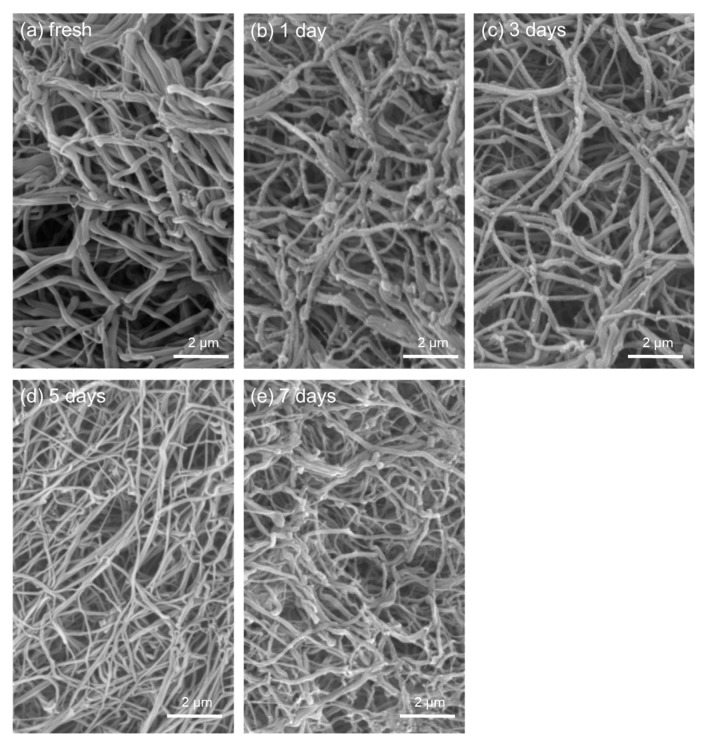
Scanning electron microscopy (SEM) images of fibrin fibers formed in PRFMs prepared from WB samples stored for the indicated periods. WB samples were simultaneously collected from the same donor. Similar findings were obtained in three other experiments.

Storage time did not substantially affect the visual appearance, size, or serum retention of PRFMs prepared from stored WB samples (Figure 5). However, fibrin fibers formed in these PRFMs became somewhat thinner with time (Figure 6).

**Figure 7:**
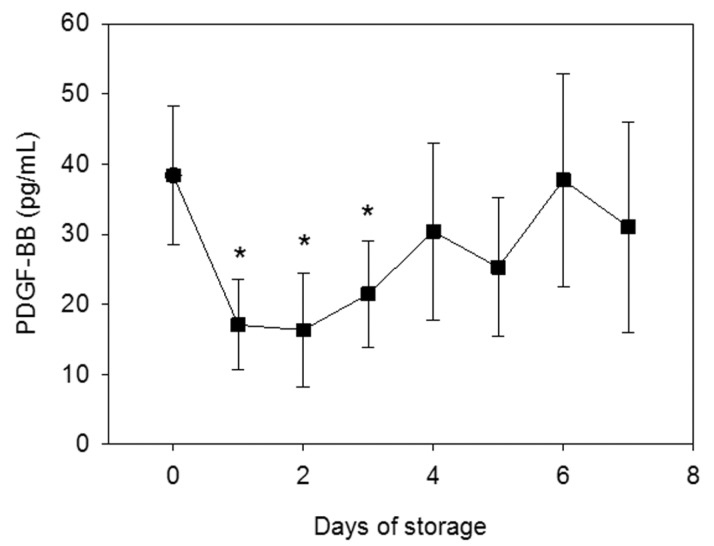
Time-dependent changes in the concentration of PDGF-BB extracted from PRFM samples that were prepared from stored WB samples and compressed to squeeze out PRFM exudates. * *p* < 0.05 as compared with fresh WB samples as controls (*n* = 8).

PDGF-BB levels in the extracts of the resulting PRFM samples significantly decreased during the initial 3 days but recovered to control levels thereafter (Figure 7).

## 4. Discussion

The biochemical mechanisms underlying different phases of platelet activation, including adhesion, shape change, the granule release reaction, and aggregation, have been well delineated [18]. To treat specific diseases, such as thrombocytopenia, functionally complete platelets are required. However, in the PRFM used for regenerative therapy, platelets are only required to aggregate in response to Ca^2+^ and/or thrombin, to release growth factors, and to support clot formation. In our previous study [7], we demonstrated that short-term storage does not influence the minimally required platelet functions or quality of the PRFM. The aim of this study was to investigate the possible expiry limit for the storage of PRFM. In general [19], the storage of platelets for clinical use is limited to a maximum of 5 days. Consequently, we did not extend the storage period to >7 days, and we assessed the quality of PRFMs prepared from stored WB samples.

Both RBC and WBC counts tended to gradually, but not significantly, decrease with storage time, whereas platelet numbers did not. Regarding the time-dependent changes in Ca^2+^, glucose, and PDGF-BB levels, as previously demonstrated [7], PDGF-BB levels in PRFMs prepared from WB samples stored for 1–3 days were significantly lower than those of fresh WB samples. Nonetheless, with increasing storage time, PDGF-BB levels recovered to those of the freshly prepared PRFM. Only glucose levels changed with time; they decreased with increasing storage time, and at 5 days and later, they were significantly lower than those of the ACD-treated WB samples on day 1.

It is generally accepted that adequate oxygen supply is needed to increase platelet viability in platelet concentrates because oxygen reduces their glucose consumption and lactate production [20,21]. It is known that even under the improved storage conditions, platelets gradually lose their function, a phenomenon that is called the storage lesion [22]. Furthermore, it was recently demonstrated that growth factors in PRP degrade in the course of storage at 22 °C [23]. Therefore, Bausset et al. recommend injecting PRP within 3 h after preparation to avoid the loss of efficacy [24]. There is an opposite viewpoint, that PRP for tissue regeneration can be stored for at least 5 days [25].

In the present study, gas-impermeable plastic tubes were used for WB preservation, so that blood cell viability can be maintained mainly by glycolysis (as explained elsewhere [26,27]) of glucose provided by the ACD-A solution [26,27] after depletion of remaining oxygen. In this case, even though platelets are not concentrated as highly as platelet concentrates for storage, it is possible that RBCs in cooperation with platelets produce lactate and significantly decrease pH. On the other hand, plasma pH of the stored WB samples remained constant at ~6.5 under our preservation conditions. As a result, platelets could be preserved well, judging by the finding that the ability of the platelets—isolated from 7-day-stored WB samples—to respond to Ca^2+^ and ADP challenges was mostly similar to that of platelets obtained from the WB samples following short-term storage. A possible explanation for this successful preservation may be suppression of cell metabolism by citrate-dependent Ca^2+^ chelation: platelet activation is known to be prevented by citrate [28], whereas it is also possible that RBC activity can be reduced through inhibition of Ca^2+^-mediated cellular functions [29] by Ca^2+^ chelation.

Because WBC-depleted transfusion is necessary to avoid WBC-mediated adverse reactions, particularly in allogeneic blood transfusions [30], the lifespan of WBCs in vitro has not been clearly described in the literature: approximate lifespans of circulating RBCs, platelets, neutrophils, eosinophils, and B cells are reported to be 120, 10, 1–5, and 2–5 days and 4–7 weeks, respectively [31]. Accordingly, granulocytes (large components of WBCs) may have the shortest survival period in vitro. In contrast, lymphocytes, which constitute the small components of WBCs, and RBCs may survive longer than can other blood cell types in vitro. Consistent with these standard lifespans, our present findings indicate that the percentage of large WBCs decreased with time. Although WBC counts did not significantly decrease within 7 days, it cannot be ruled out that the automatic hematology analyzer, which uses particle size differences for calculations, may have detected and counted WBCs that were reduced in size, probably by apoptosis.

The thickness of fibrin fibers of PRFM samples gradually decreased with time, probably due to degradation of coagulation factors, a reduction in their enzymatic activity, or a decline of platelet functions. Therefore, it can be hypothesized that PRFMs composed of thinner fibrin fibers may be easily susceptible to degradation and may release growth factors faster than do fresh WB samples. Nevertheless, fibrin fibers observed in fibrin clots that were prepared from fresh or frozen platelet-poor plasma by the addition of thrombin were considerably thinner, and their cross-link density was considerably higher relative to stored WB samples [7]. In our preliminary study, with a limited number of WB samples, the degradation assay involving a trypsin and EDTA solution failed to detect significant differences in PRFMs prepared from long-term–stored and fresh WB samples (data not shown). We believe that the PRFM prepared from WB samples stored long-term can be an alternative option for regenerative therapy in clinical settings.

Although blood transfusion studies have established standard protocols for the storage of WB samples [32,33], the time-dependent reduction in WBC counts may result in weakened bactericidal effects [34]. In addition, the possibility that autolysis of WBCs can trigger degradation of specific proteins, which in turn can influence PRFM formation, cannot be ruled out. Consequently, to validate the clinical use of such a PRFM, its safety and efficacy should be assessed further in experimental models based on relatively large animals.

## 5. Conclusions

The PRFM is conventionally prepared onsite; however, it would be convenient if this material were prepared several days later. We demonstrated that a clinically applicable PRFM can be prepared from WB samples that are stored for up to 7 days by the addition of appropriate amounts of Ca^2+^. This method can make the treatment schedule more flexible and benefit both the patients and physicians or dentists involved in regenerative therapy with the PRFM. Although the period of WB sample storage may be further extended by improving several conditions, we would recommend using a fresh autologous PRFM prepared onsite as the first choice and the PRFM prepared from stored autologous WB samples as the second choice. To minimize the possible loss of efficacy and unidentified or unpredictable risks, it would be better to utilize the stored autologous WB samples as soon as possible, at least within a week in accordance with the national guidelines [6].
